# Asymmetry matters: A genomic assessment of directional biases in gene flow between hybridizing spruces

**DOI:** 10.1002/ece3.2682

**Published:** 2017-04-22

**Authors:** Guillaume de Lafontaine, Jean Bousquet

**Affiliations:** ^1^ Canada Research Chair in Forest GenomicsCentre for Forest Research and Institute of Systems and Integrative BiologyUniversité LavalQuébecQCCanada; ^2^ Department of Plant BiologyUniversity of IllinoisUrbanaILUSA

**Keywords:** introgression, *Picea mariana*, *Picea rubens*, reproductive barriers, single nucleotide polymorphisms

## Abstract

Assessing directional bias in interspecific gene flow might be important in determining the evolutionary trajectory of closely related species pairs. Using a set of 300 single nucleotide polymorphisms (SNPs) having variable propensity to cross species boundary, we evaluated the genomic extent and direction of interspecific gene flow in a progenitor‐derivative spruce species pair (black spruce and red spruce). A higher rate of gene flow was found from black spruce toward red spruce purebreds than vice versa. This asymmetry could reflect the historical gene flow between the two taxa at the time of species inception and during postglacial colonization. A clear asymmetry in introgression was depicted by a greater gene flow between red spruce and hybrids than between black spruce and hybrids. While backcrossing toward red spruce was invariably high across the genome, the actual species boundary is between hybrids and black spruce where gene flow is impeded at those genomic regions impermeable to introgression. Associations between hybrid index and climatic variables (total annual precipitation and mean annual temperature) were tested, as these might indicate a role for exogenous selection in maintaining the species boundary. While an apparent association was found between the hybrid index and precipitation, it collapsed when considered in light of the directional bias in interspecific gene flow. Hence, considering asymmetrical patterns of introgression allowed us to falsify an apparent role for exogenous selection. Although this was not formerly tested here, we suggest that this pattern could result from asymmetrical endogenous selection, a contention that deserves further investigations.

## 1 Introduction

Natural interspecific hybridization and introgressive hybridization (i.e., introgression) are ubiquitous in plants and have played a prominent role in their evolution (Anderson, 1949; Arnold, 1997). Interspecific gene flow is often asymmetrical in plants and might result in directional patterns of introgression (Arnold, Tang, Knapp, & Martin, 2010; Bacilieri, Ducousso, Petit, & Kremer, 1996; De La Torre, Ingvarsson, & Aitken, 2015; Guichoux et al., 2013; Lowry, Modliszewski, Wright, Wu, & Willis, 2008; Petit, Bodénès, Ducousso, Roussel, & Kremer, 2003; Starr, Gadek, Yoder, Flatz, & Smith, 2013; Tiffin, Olson, & Moyle, 2001). Causal factors that have been invoked for such asymmetrical interspecific gene flow include differences in phenology, fertilization success, and offspring viability (Baack, Melo, Rieseberg, & Ortiz‐Barrientos, 2015; Carney, Cruzan, & Arnold, 1994; Field, Ayre, Whelan, & Young, 2011; Keim, Paige, Whitham, & Lark, 1989; Rieseberg & Blackman, 2010; Starr et al., 2013; Sweigart & Willis, 2003), differences in selective advantages under certain environmental conditions (De La Torre et al., 2015; Lexer, Fay, Joseph, Nica, & Heinze, 2005; Martin, Bouck, & Arnold, 2006), differences in species abundance at the time of mating (Burgess, Morgan, Deverno, & Husband, 2005; Lepais et al., 2009), or contrasted population dynamics related to range shifts and expansions (Currat, Ruedi, Petit, & Excoffier, 2008; Guichoux et al., 2013). Assessing directional biases in interspecific gene flow and their driving processes might be important in determining the evolutionary trajectory of closely related species pairs (Fitzpatrick et al., 2010; Petit et al., 2003; Rhymer & Simberloff, 1996).

The notion that interspecific gene flow and reproductive isolation are characteristics of genome regions, and not entire genomes (Key, 1968; Wu, 2001), has recently gained much interest with the advent of genomic methods to analyze large numbers of loci (Lexer & Widmer, 2008; Strasburg et al., 2012). Indeed, recent population genomic studies exploring locus‐level differences between closely related species have revealed that heterogeneous patterns of introgression across the genome are commonplace in the natural populations of various taxa (Harrison & Larson, 2014; Kane et al., 2009; Luttikhuizen, Drent, Peijnenburg, van der Veer, & Johannesson, 2012; Minder & Widmer, 2008; Nolte, Gompert, & Buerkle, 2009; Rieseberg, Whitton, & Gardner, 1999) including trees (Hamilton, Lexer, & Aitken, 2013a; de Lafontaine, Prunier, Gérardi, & Bousquet, 2015; Lexer et al., 2010; Martinsen, Whitham, Turek, & Keim, 2001; Scotti‐Saintagne et al., 2004). Specifically, all these studies support the view that some key genomic regions remain virtually impermeable to interspecific gene flow while most of the genome can be exchanged somewhat freely between species.

So far, most studies that have investigated asymmetrical patterns of interspecific gene flow have relied on a modest number of presumably neutral markers, under the assumption that these should reflect the species' entire genome, thus conceived as a cohesive unit. However, just as the overall level of introgression is heterogeneous across the genome, the level of asymmetry in interspecific gene flow might also be variable across the genome (Fitzpatrick et al., 2010). We might specifically expect that the directional bias in introgression should be stronger at less permeable genomic regions, that is, at these key loci where gene flow is actually impeded between species (Currat et al., 2008). Accordingly, asymmetrical introgression between European oak species (*Quercus robur* and *Quercus petraea*) was reported when analyzing a subset of 60 interspecific outlier loci (from an *F*_ST_‐outlier analysis) but not when using a presumably selectively neutral subset of 166 nonoutlier markers (Guichoux et al., 2013).

**Figure 1:**
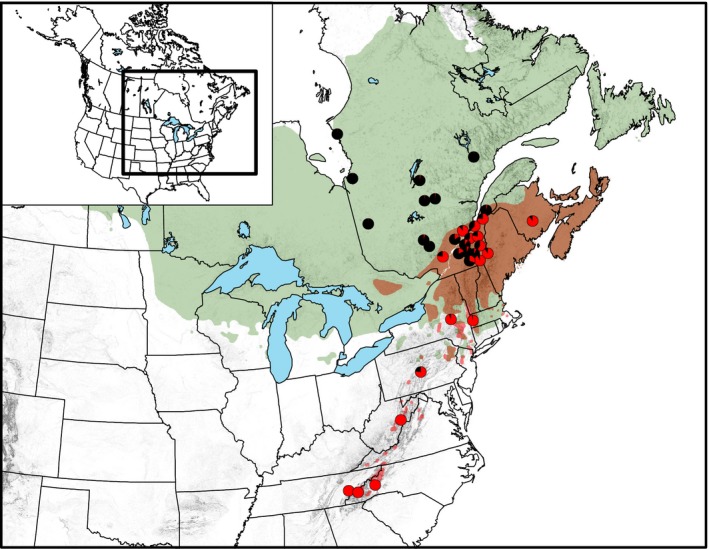
Sampling of 33 populations across the contact zone between *Picea mariana* and *Picea rubens* in eastern North America. Ranges of *P. mariana* and *P. rubens* are shown in green and red, respectively, with the sympatric area in brown. Pie charts indicate mean genomic proportion of *P. mariana* (black) and *P. rubens* (red) ancestry in each sampled population based on 300 SNPs using the Bayesian clustering algorithm implemented in STRUCTURE (*K *=* *2)

Black spruce (*Picea mariana* [Mill] B.S.P.) and red spruce (*Picea rubens* Sarg.) are two closely related North American conifers thought to have a fairly recent speciation history (Jaramillo‐Correa & Bousquet, 2003; Perron, Perry, Andalo, & Bousquet, 2000). Red spruce likely originated from the glaciation‐induced isolation of a preexisting black spruce population during the Pleistocene (i.e., allopatric speciation). The two taxa would have then come into secondary contact during the Late Holocene (Lindbladh, Jacobson, & Schauffler, 2003; Perron et al., 2000). The current natural range of black spruce spans the transcontinental boreal forest of North America whereas red spruce has a narrow distribution in the montane forests along the Appalachian Mountains of the United States, the Acadian forest of the Maritime Provinces of Canada, and the mixed forest of southern Québec where the climate is typically more humid and temperate. The two taxa co‐occur in sympatry over a large geographic area in northeastern North America where their ranges overlap (Figure 1). Frequent occurrences of hybrid trees have been reported within the contact zone along the St. Lawrence River valley in Southern Québec, testifying of the extensive gene flow between the two spruce taxa (de Lafontaine et al., 2015; Morgenstern & Farrar, 1964; Perron & Bousquet, 1997).

It is generally recognized that the maintenance of species integrity in the face of interspecific gene flow can be driven by exogenous or endogenous selective pressures (i.e., ecological selection versus selection against heterozygotes or Bateson–Dobzhansky–Muller incompatibilities; Arnold, 1997; Barton & Hewitt, 1985; Moore, 1977; Nosil, 2012). Attempts at disentangling the roles of environment‐dependent and environment‐independent factors in driving patterns of introgression between black spruce and red spruce have given mixed results. On the one hand, transplant experiments have shown that red spruce is more shade‐tolerant but less cold and drought‐tolerant than black spruce, and that black spruce has higher allocation to stem wood, while red spruce has higher allocation to roots (Major, Barsi, Mosseler, Campbell, & Rajora, 2003; Major, Mosseler, Barsi, Campbell, & Rajora, 2003a,b; Manley & Ledig, 1979). These results suggest that the two taxa are ecologically distinct, each exhibiting physiological traits that favor different ecological niches. Red spruce has a greater competitive advantage in warm and mesic conditions while black spruce outperforms in cold and dry environments. Hence, these observations suggest a role for exogenous selection whereby interspecific gene flow is impeded as a result of different selective advantages under contrasted levels of ecological (i.e., moisture and temperature) conditions. On the other hand, controlled crosses suggested that endogenous selective pressures might also be important drivers of hybridization and introgression. Indeed, first‐generation hybrids had lower fitness, as illustrated by higher rates of empty and aborted seeds, lower germination rates, lower CO_2_ uptake, and lower juvenile survival (Major et al., 2003b, 2005; Manley & Ledig, 1979). No record of reproductive phenological barrier between these closely related species is reported in the literature.

Heterogeneous patterns of interspecific gene flow have recently been uncovered across the genome of hybridizing black spruce and red spruce (de Lafontaine et al., 2015). In this previous study, 300 single nucleotide polymorphism (SNP) loci spanning the 12 chromosomes of black spruce were grouped into three classes of markers on the basis of their increasing capacity to cross the boundary between the two spruce taxa (impermeable, neutral, and highly permeable marker subsets, respectively) indicating variable states of introgression along the genome. The main objective of the present study was to assess whether interspecific gene flow between *P. mariana* and *P. rubens* is asymmetrical, and if so, whether the level of asymmetry is heterogeneous across the genome. We expected that the directional bias in introgression will vary across the three classes of markers as a result of the differential outcome of historical, demographic, and natural selection processes on the three marker subsets (Guichoux et al., 2013). As a complementary objective, we explored the putative role of ecological (exogenous) selective pressures in maintaining the hybrid zone and the integrity of species in the face of introgression. Because there is no possibility to replicate samples of the studied hybrid zone in different settings (as in Lexer et al., 2010), the geographic context of the hybrid zone between black spruce and red spruce does not lend itself easily to a direct assessment of the relative importance of exogenous versus endogenous (genetic) selective processes (see de Lafontaine et al., 2015). Hence, while formally disentangling the role of environmental and genetic factors in the maintenance of this hybrid zone remains beyond the scope of this study, we did hypothesize that uncovering directional biases in introgression could perhaps provide further useful insights regarding the relative importance of ecological selective pressures. Specifically, given that these two taxa are associated with contrasted ecological niches (Major, Barsi, et al., 2003; Major et al., 2003a,b; Manley & Ledig, 1979), we expected that variation in environmental conditions should reflect the extent and direction of introgression if ecological selection is an important determinant of the genetic boundary between species.

## 2 Materials and Methods

A total of 385 adult individual trees from 33 populations spanning the allopatric and sympatric zones of *P. mariana* and *P. rubens* (Figure 1 and Table S1) were successfully genotyped at 300 SNP loci representing 279 genes spread over the 12 chromosomes of black spruce (de Lafontaine et al., 2015; Dryad repository DOI:10.5061/dryad.9kb02). The intersection of at least two of three population genomic approaches (namely *F*_ST_‐outlier analysis, geographic, and genomic clines) was used to classify the sampled loci according to their capacity to introgression. Of the 300 SNPs, 23 were classified as impermeable to introgression, 238 were assumed to be neutral, and 39 were highly permeable to interspecific gene flow (de Lafontaine et al., 2015). Here, we assessed the direction of interspecific gene flow estimated from each of the three marker subsets and to evaluate whether variation in environmental conditions reflects the direction of introgression. Detailed methods regarding SNP discovery, DNA extraction, and genotyping can be found elsewhere (de Lafontaine et al., 2015).

### 2.1 Detection of admixed individuals

The proportion of black spruce and red spruce genome admixture for each sampled individual (hybrid index; *q*‐value) was estimated based on all SNPs, using the Bayesian clustering algorithm implemented in STRUCTURE 2.3.3 (Pritchard, Stephens, & Donnelly, 2000). Five independent runs, each with 1,000,000 Markov chain Monte Carlo (MCMC) iterations after 50,000 burn‐in periods, were performed for *K* (number of groups) set to 2, which corresponded to the optimal partitioning (de Lafontaine et al., 2015) and to the actual number of taxa. Expected levels of admixture are 0 and 1 for purebreds, 0.5 for first‐generation (F_1_) hybrids, and 0.25 and 0.75 for backcrossed individuals. Hence, theoretically, threshold values of 0.125 and 0.875 should be optimal for distinguishing between purebreds and first‐generation backcrossed individuals. We used simulations to assess the efficiency (proportion of individuals in a category that are correctly identified) and accuracy (proportion on an identified category that truly belongs to that category) of the STRUCTURE analysis to tease apart purebreds from admixed individuals (Vähä & Primmer, 2006). Simulated *P. mariana*,* P. rubens*, first‐generation hybrids (F_1_) and backcrosses with each parental (F_1_ × *P. mariana* and F_1_ × *P. rubens*) were obtained using HYBRIDLAB 1.0 (Nielsen, Bach, & Kotlick, 2006). We used the multilocus genotypes (300 SNPs) of individuals from the allopatric population group of each taxon as reference parental genotypes (*n *=* *92 and 98 individuals for *P. mariana* and *P. rubens*, respectively). Simulated *P. mariana*,* P. rubens*, F_1_, F_1_ × *P. mariana*, and F_1_ × *P. rubens* (*n *=* *1,000 simulated individuals per category) were analyzed in STRUCTURE with five runs of 500,000 MCMC iterations after 10,000 burn‐in periods.

### 2.2 Estimation of asymmetrical gene flow

The program MIGRATE‐N was originally designed and is still most typically used to estimate effective population sizes and past migration rates among n populations (i.e., movement of individuals within a metapopulation) (Beerli & Felsentein, 1999, 2001; Beerli & Palczewski, 2010). The analysis uses maximum‐likelihood or Bayesian inference to jointly estimate the parameters *M* (*M *= *m*/μ; where *m* and μ are the migration and mutation rates, respectively) describing the mutation‐scaled asymmetrical migration rate, and Θ (Θ = 4*N*_e_μ) describing the mutation‐scaled effective population size in a coalescent framework by which alleles are traced back in time to a single ancestral copy. Importantly, *Nm* in MIGRATE‐N has the same meaning as *Nm* in the context of *F*_ST_. As seen in any other population genetics method, *m* is seen as a backward immigration rate, which, in other words, is the probability that a randomly chosen individual (i.e., a multilocus genotype) in a given generation came from a population (i.e., a gene pool) different from the one in which it is currently observed in the preceding generation. In recent years, instead of assessing spatial movement of individuals among populations, an increasing number of studies have successfully used the MIGRATE‐N approach to test various scenarios of asymmetrical gene flow between closely related sympatric species and their hybrids based on STRUCTURE assignments of individuals (e.g., Andrew, Ostevik, Ebert, & Rieseberg, 2012; Field et al., 2011; Ortego, Gugger, & Sork, 2016; Scascitelli et al., 2010; Starr et al., 2013). Following this novel approach, we estimated the magnitude and direction of long‐term gene flow between the two spruce taxa and their hybrids using the maximum‐likelihood coalescent‐based approach implemented in MIGRATE‐N 3.6 (Beerli & Felsenstein, 1999, 2001). The 195 individuals from the sympatric zone (Figure 1) were assigned to one of three genotypic classes on the basis of their STRUCTURE *q*‐values: pure *P. mariana* (*q*‐values < 0.125), pure *P. rubens* (*q*‐values > 0.875), or hybrids (*q*‐values between 0.125 and 0.875). For each marker subset (impermeable, neutral, and highly permeable, *n *=* *23, 238, and 39 SNPs, respectively), we estimated Θ for each genotypic class and *M* between all pairwise classes. For each marker subset, we conducted 10 replicate runs and changed the random number seed, the starting values of Θ and *M*, and search parameters among runs. For the first run, initial values of Θ and *M* were estimated from *F*_ST,_ while for the subsequent runs, we iteratively used the mean maximum‐likelihood estimates (MLE) of Θ and *M* over the previous runs as starting parameters, as suggested by Beerli (2009). Search parameters included a first set of seven replicate runs with 10 short chains, each visiting 10,000 genealogies and using 500 trees to improve the driving values for the next chain as well as three long chains, each visiting 100,000 samplings with a burn‐in of 5,000 trees. To insure convergence, the following three runs were longer (×5) and consisted of 10 short chains of 50,000 trees, followed by three long chains of 500,000 steps, with the first 10,000 trees discarded as burn‐in. This search strategy was deemed satisfactory because the results (Θ and *M* parameter estimates) were largely congruent among runs (Beerli, 2009). However, the 95% confidence intervals generated by any individual run of MIGRATE‐N were too narrow, a caveat already reported on the basis of simulations (Abdo, Crandall, & Joyce, 2004). Hence, we used the variation across the MLE obtained from our 10 replicate runs to compute the mean and standard deviation of Θ and *M* parameter estimates. Differences among these parameters were tested by analyses of variance (ANOVAs) followed by post hoc analyses using Tukey's HSD (honest significant difference) test for multiple comparisons. False discovery rate (FDR; Benjamini & Yekutieli, 2001) control was performed to adjust *p*‐values for all multiple hypotheses tests (α = 0.05), and all reported *p*‐values were FDR‐adjusted.

### 2.3 Climatic data analysis

The latitude, longitude, and elevation of sampling locations were input into BIOSIM 10 (Régnière, 1996) which interpolates daily climatic conditions using a simulation model that integrates climatic data recorded between 1970 and 2000 at the four closest weather stations of each sampling location within a range of 50 km. Nine climatic variables were initially assessed: the annual mean temperature (°C), the annual mean of the daily minimal temperature, the annual mean of the daily maximal temperature, the annual number of degree‐days above 0°C, above 5°C and above 10°C, the number of frost days, the total annual amount of precipitation (in mm of water including rain and snow), and the annual amount of snow (in mm of water). All climatic variables were highly correlated with either mean annual temperature or total annual amount of precipitation (as previously reported in Prunier, Gérardi, Laroche, Beaulieu, & Bousquet, 2012); hence, only these two latter climatic factors were retained for further analysis (Table S1). In order to assess whether variation in environmental conditions reflects the direction of introgression, significant differences in the mean of temperature and precipitation variables among geographic/genotypic classes (i.e., allopatric *P. mariana*, sympatric *P. mariana*, hybrids, sympatric *P. rubens*, allopatric *P. rubens*) were tested by analysis of variance (ANOVA) followed by post hoc analysis using Tukey's HSD (honest significant difference) test for multiple comparisons along with FDR control. Furthermore, the relationship between the hybrid index (*q*‐value) and each climatic variable within the sympatric zone was tested with linear least‐squares regression.

## 3 Results

### 3.1 Detection of admixed individuals

**Figure 2:**
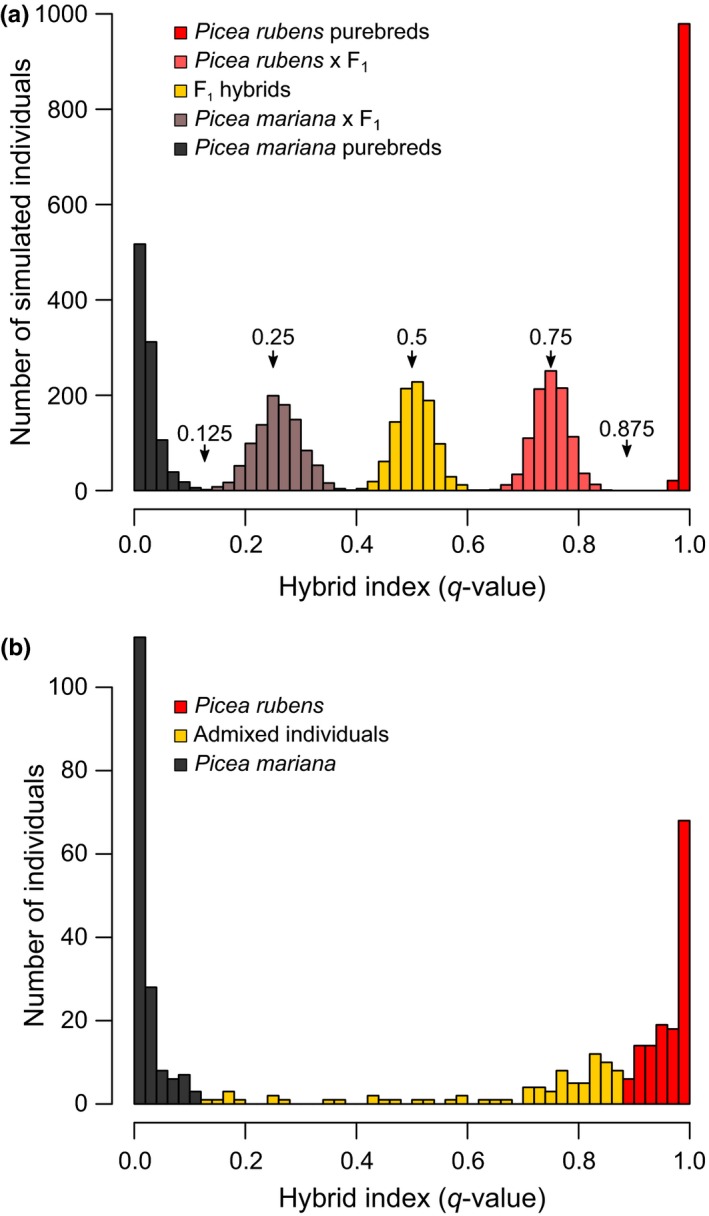
Frequency distribution of hybrid indexes (STRUCTURE *q*‐values) of multilocus genotypes (*n *=* *300 SNP loci) of (a) simulated purebreds, first‐generation backcrosses, and first‐generation (F_1_) hybrid individuals (*n *=* *1,000 simulated individuals per category) and (b) adult spruce tree individuals (*n *=* *385 individuals) sampled across the allopatric and sympatric zones of *Picea mariana* and *Picea rubens*. Sampled spruce tree individuals were assigned to one of three genotypic classes on the basis of their STRUCTURE *q*‐values: pure *P. mariana* (*q*‐values < 0.125), pure *P. rubens* (*q*‐values > 0.875), or admixed individuals (i.e., hybrids sensu lato; *q*‐values between 0.125 and 0.875)

Simulations were used to assess the efficiency (proportion of individuals in a category that are correctly identified) and accuracy (proportion on an identified category that truly belongs to that category) of the STRUCTURE analysis to distinguish purebreds from admixed individuals (i.e., hybrids sensu lato) using the theoretical threshold hybrid indexes (*q*‐values of 0.125 and 0.875; see Materials and methods). STRUCTURE assignments of simulated individuals based on all 300 SNPs resulted in almost complete lack of overlap among the five categories assayed (simulated *P. mariana*, F_1_ × *P. mariana*, F_1_, F_1_ × *P. rubens*, and *P. rubens*; Figure 2a). Hence, simulation results indicated high accuracy and efficiency of the STRUCTURE analysis (both values above 99.8%) to tease apart purebreds from first‐generation backcrosses using the theoretical thresholds (Figure 2a).

Based on these thresholds, assignment scores of the actual 385 sampled spruce trees indicated that 21% were admixed individuals (Figure 2b). Within the sympatric zone, the proportion of hybrids increased to 38%. While these admixed individuals belong to a wide range of hybrid generations, the distribution of the hybrid indexes was clearly a skewed (Figure 2b). Specifically, 74% of admixed individuals were backcrossed toward red spruce. The remaining hybrids were distributed at low frequency across the various levels of admixed ancestries, including recent‐generation hybrids (11%) and backcrosses toward black spruce (11%), along with advanced‐generation hybrids falling in between the simulated categories (4%). In practice, the individuals assigned as purebreds by STRUCTURE analysis (79% of total sample) should indeed include mostly pure *P. mariana* or *P. rubens* individuals, as well as some late‐generation backcrosses toward the parental taxa.

### 3.2 Estimation of asymmetrical gene flow

**Figure 3:**
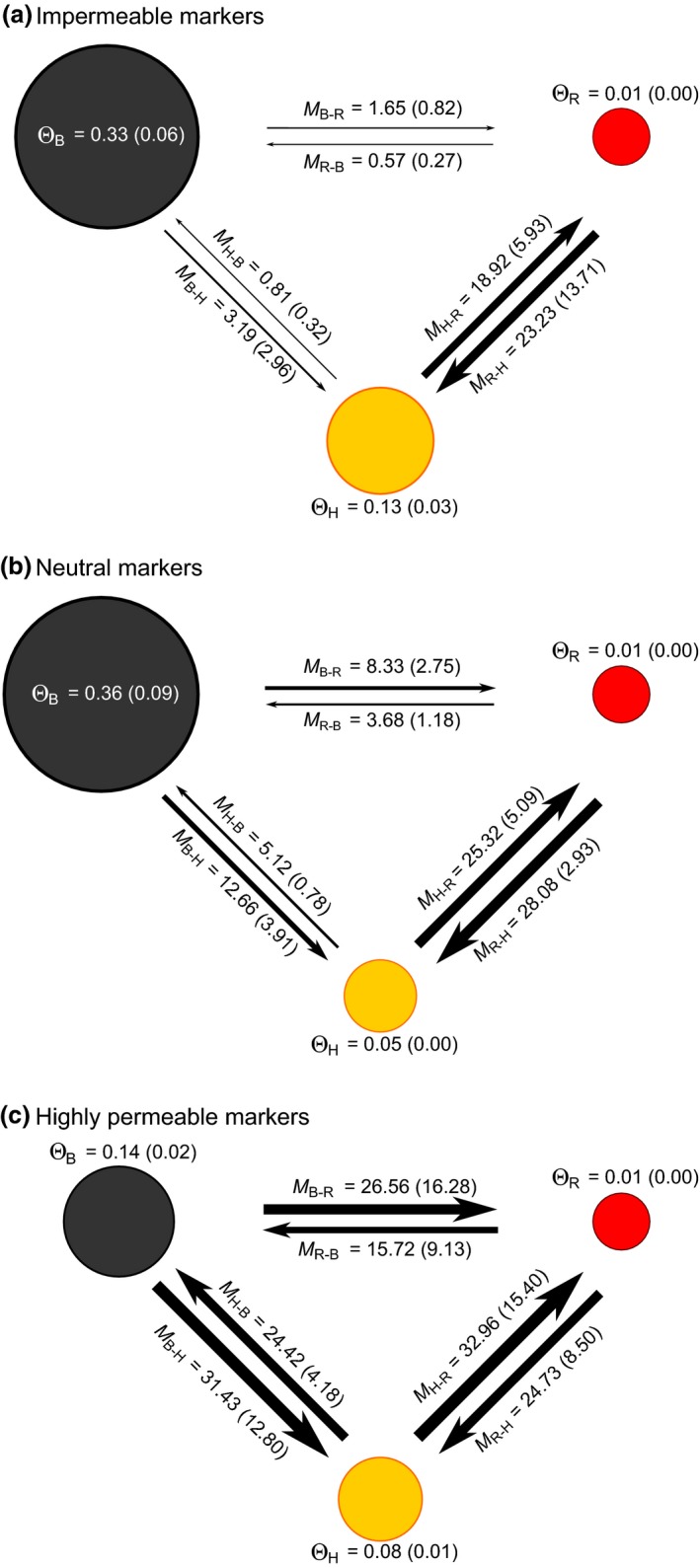
Mean maximum‐likelihood estimates (standard deviation in parentheses) of the mutation‐scaled effective population size (Θ) and the mutation‐scaled migration rate (*M*) among *Picea marina* (_B_), *Picea rubens* (_R_), and hybrids (_H_) (represented by dark gray, red, and yellow circles, respectively) in the sympatric zone. Parameter estimates were computed over 10 replicate MIGRATE‐N runs for (a) a set of markers impermeable to interspecific gene flow (*n *=* *23 SNP loci), (b) a set of neutral markers (*n *=* *238 SNP loci), and (c) a set of markers highly permeable to interspecific gene flow (*n *=* *39 SNP loci). Circle size and thickness of arrows proportional to parameter estimates

The coalescent‐based approach implemented in MIGRATE‐N was used to jointly estimate the effective population size as well as the magnitude and direction of gene flow between the two spruce taxa and their hybrids within the sympatric zone. Results of this analysis are summarized in Figure 3 where means and standard deviations of Θ and *M* parameter estimates (see Section 2) are reported while MLE along with their 95% confidence intervals for each MIGRATE‐N run are available in Table S2. For all three sets of markers, the mutation‐scaled effective population size (Θ) was significantly higher in black spruce, intermediate in hybrids, and lower in red spruce (all *p*‐values <.0001), a result consistent with differences in geographic ranges (Figure 1). The mutation‐scaled migration rates (*M*) between *P. mariana* and *P. rubens* significantly increased in both directions (*M*_*P.mariana*↔*P.rubens*_) among the marker subsets according to their increasing capacity to cross the boundary between the two spruce taxa (i.e., negligible gene flow at impermeable loci, intermediate levels at presumably neutral loci, and high gene flow at highly permeable loci; *p*‐value <.0001). There was a higher rate of unidirectional gene flow from black spruce to red spruce than the opposite (*M*_*P.mariana*→*P.rubens*_ > *M*_*P.rubens*→*P.mariana*_). This asymmetry was significant with the impermeable and neutral markers subset (*p*‐values = .002 and.0006, respectively) but not with highly permeable markers (*p*‐value = .15). Bidirectional gene flow was high between red spruce and hybrids (*M*_*P.rubens*↔*Hybrids*_) and did not vary significantly among the three marker subsets (*p*‐values = .07). By contrast, bidirectional gene flow between black spruce and hybrids (*M*_*P.mariana*↔*Hybrids*_) increased among the marker subsets with negligible gene flow at impermeable loci, intermediate levels at presumably neutral loci, and high gene flow at highly permeable loci (*p*‐value < .0001). Hence, there was a clear asymmetry in introgression with greater overall (bidirectional) gene flow between red spruce and hybrids than between black spruce and hybrids (*M*_*P.rubens*↔*Hybrids*_ > *M*_*P.mariana*↔*Hybrids*_; *p*‐value < .0001). However, no significant unidirectional gene flow asymmetry was found between purebreds and hybrids (i.e., *M*_*P.mariana*→*Hybrids*_ = *M*_*Hybrids* →*P.mariana*_; *M*_*P.rubens*→*Hybrids*_ = *M*_*Hybrids* →*P.rubens*_; all *p*‐values ≥.24), except between black spruce and hybrids at neutral markers (*M*_*P.mariana*→*Hybrids*_ > *M*_*Hybrids*→*P.mariana*_; *p*‐value = .002). In summary, at impermeable markers, there was high gene flow between red spruce and hybrids but negligible gene flow between black spruce and red spruce as well as between black spruce and hybrids (*M*_*P.rubens*↔*Hybrids*_ > *M*_*P.mariana*↔*P.rubens*_ = *M*_*P.mariana*↔*Hybrids*_; *p*‐value < .0001). A similar scenario was found at presumably neutral markers where there was high gene flow between red spruce and hybrids but intermediate gene flow between black spruce and red spruce as well as between black spruce and hybrids (*M*_*P.rubens*↔*Hybrids*_ > *M*_*P.mariana*↔*P.rubens*_ = *M*_*P.mariana*↔*Hybrids*_; *p*‐value < .0001). However, at highly permeable markers, the level of gene flow was uniformly high among all three genotypic classes (*M*_*P.rubens*↔*Hybrids*_ = *M*_*P.mariana*↔*P.rubens*_ = *M*_*P.mariana*↔*Hybrids*_; *p*‐value = .20).

### 3.3 Climatic data analysis

**Figure 4:**
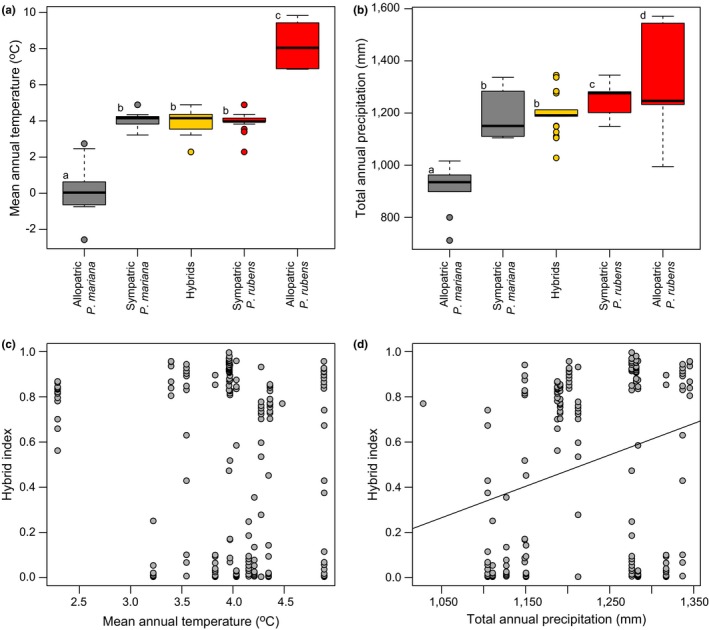
Variation in mean annual temperature and total annual precipitation among geographic and genotypic classes (a,b) and scatter plots showing the relationship between hybrid index (STRUCTURE *q*‐value; 0 = *Picea mariana*, 1 = *Picea rubens*) and each climatic variable in the sympatric zone (c,d). Boxplots indicate lower quartile, median, and upper quartile. Whiskers' length is 1.5× interquartile range. Significant differences in mean values are indicated by different lowercase letters

Significant differences in both temperature and precipitation variables were found among the various geographic and genotypic classes (both *p*‐values < .0001; Figure 4a,b). Mean annual temperature was significantly lower in the black spruce allopatric zone, intermediate in the sympatric zone, and higher in the allopatric red spruce zone (Figure 4a). The trend was roughly similar for total annual precipitation. However, within the sympatric zone, on average, red spruce individuals received more precipitation than both hybrids and black spruce individuals; that is, at a level not significantly different from that of the red spruce allopatric zone (Figure 4b). A closer look at the relationship between hybrid index and each climatic variable within the sympatric zone corroborates these results. While the hybrid index did not vary according to mean annual temperature (*p*‐value = .21; Figure 4c), there was a significant increase in *P. rubens* ancestry with increasing amount of total annual precipitation, although the explanatory power of this linear model was quite low (*p*‐value = 0.0001, *r* ^2^ = 0.07; Figure 4d).

## 4 Discussion

Uncovering the patterns of gene flow between closely related taxa is important for understanding the processes that maintain distinct species. Studies reporting morphological identifications of red spruce and black spruce and molecular assessments of the extent of introgression between these taxa have been quite controversial (Bobola et al., 1996; Gordon, 1976; Manley, 1972; Perron & Bousquet, 1997). Yet, in the present study, simulations indicated that the STRUCTURE clustering analysis used with a set of 300 SNP markers allowed to tease apart purebreds from first‐generation backcrosses using the theoretical thresholds with high accuracy and efficiency (Vähä & Primmer, 2006). Using this approach, the proportion of admixed individuals (i.e., hybrids sensu lato) reached 38% within the sympatric zone, supporting previous morphological and genetics studies reporting extensive interspecific gene flow between black spruce and red spruce (Morgenstern & Farrar, 1964; Perron & Bousquet, 1997). However, the level of introgression between these two spruce taxa is not homogeneous across the genome and, more specifically, gene flow varied among the 300 markers assayed in the present study (de Lafontaine et al., 2015). This property was further explored here in order to assess whether the level of asymmetry in interspecific gene flow between *P. mariana* and *P. rubens* is also heterogeneous across the genome.

Within the sympatric zone, there was a higher rate of unidirectional gene flow from black spruce to red spruce purebreds than the opposite (*M*_*P.mariana*→*P.rubens*_ > *M*_*P.rubens*→*P.mariana*_). Such asymmetry, based solely on data from purebred individuals, could reflect the current different population sizes of the two taxa. According to Burgess et al. (2005), higher gene flow should be expected from the more abundant species (black spruce) to the rare species (red spruce). However, under this density‐dependant scenario, we should also expect that first‐generation hybrids will be more likely to mate with the more abundant species (Lepais et al., 2009), which, in this instance, would lead to increased directional introgression toward black spruce. Because we did not observe such a pattern and also because MIGRATE‐N delivers long‐term migration estimates (Beerli, 2009), we conclude that the migration rate between pure types should reflect the general trend in historical gene flow between the two taxa at the time of species inception as well as during postglacial colonization (Currat et al., 2008; Guichoux et al., 2013). First, red spruce originated from a glaciation‐induced isolation of a preexisting black spruce population most likely during the Pleistocene (Perron et al., 2000). As a consequence, the genetic diversity of red spruce at the DNA level is essentially a subset of that of black spruce (Jaramillo‐Correa & Bousquet, 2003; Perron et al., 2000). Hence, given the progenitor‐derivative nature of this species pair, the direction of gene flow during the speciation was necessarily higher from black spruce (progenitor) toward red spruce (derivative) than vice versa. Second, based on pollen paleorecords of eastern North America, Lindbladh et al. (2003) suggested the replacement of a black spruce forest by a red spruce‐dominated forest during the Late Holocene (since ca. 1,500 cal year BP), when red spruce massively invaded the area. According to the theoretical neutral model of introgression during range expansions (Currat et al., 2008), introgression occurs almost exclusively from the resident to the invading species. Our genetic data corroborate this scenario for the two spruce species whereby the genome of the resident taxa (black spruce) infiltrated the genomic background of the colonizing taxa (red spruce) more than the opposite. Another prediction of the neutral model of introgression is that the asymmetrical pattern of introgression from resident to invasive taxa should be strongest at markers experiencing reduced gene flow (Currat et al., 2008; Guichoux et al., 2013). Correspondingly, the asymmetry of gene flow between black spruce and red spruce was found at both impermeable and presumably neutral markers, but not at highly permeable markers, where gene flow appeared virtually unimpeded in both directions.

The most striking pattern is a clear asymmetry in introgression depicted by a greater overall (bidirectional) gene flow between red spruce and hybrids than between black spruce and hybrids (*M*_*P.rubens*↔*Hybrids*_ > *M*_*P.mariana*↔*Hybrids*_). As expected, this asymmetry was heterogeneous across the genome, but this heterogeneity was entirely due to the genomic variability of the gene flow between black spruce and hybrids. Indeed, while gene flow between red spruce and hybrids (i.e., backcrossing toward *P. rubens*) remained invariably high even for impermeable loci subset, there was an increase of total gene flow between black spruce and hybrids (i.e., backcrossing toward *P. mariana*) according to the permeability of the marker subsets (i.e., negligible gene flow at impermeable loci, intermediate levels at presumably neutral loci, and high gene flow at highly permeable loci). At highly permeable loci, the asymmetrical pattern of introgression was no longer detected, that is, at these loci, backcrossing was equally high toward both parental species.

We have argued above that the directional gene flow observed between the two parental species (black spruce toward red spruce) might reflect the known long‐term demographic history of the two spruce taxa (i.e., at the time of species inception and during postglacial colonization). Yet, it has been suggested that both historical and contemporary processes can act in concert to influence and maintain patterns of introgression (Abbott et al., 2013; Hamilton & Aitken, 2013). The higher level of backcrossing toward *P. rubens* than toward *P. mariana* might reflect more contemporary evolutionary processes including exogenous or endogenous selection. Indeed, in natural plant populations, it is commonly assumed that selection remains the primary mechanism implicated in determining contemporary patterns of hybridization and introgression (Fitzpatrick et al., 2010; Hamilton & Aitken, 2013; Hamilton et al., 2013a; Lexer et al., 2005; Martin et al., 2006; Scascitelli et al., 2010). Because red spruce and black spruce are associated with contrasted ecological niches (Major, Barsi, et al., 2003; Major et al., 2003a,b; Manley & Ledig, 1979) and as there is no evidence of reproductive phenological barrier, we expected that variation in environmental conditions should reflect the extent and direction of introgression if exogenous selection is a key determinant for the maintenance of the boundary between species.

Overall, both temperature and precipitation variables (mean annual temperature and total annual amount of precipitation) had higher values for red spruce than for black spruce, with intermediate values for hybrids. These results are largely congruent with transplant experiments suggesting that red spruce has a greater competitive advantage in warm and mesic conditions while black spruce outperforms in cold and dry environments (Major, Barsi, et al., 2003; Major et al., 2003a,b; Manley & Ledig, 1979). On this basis, it could be tempting to argue that exogenous selection might have played a role in the maintenance of species integrity, with parental species locally adapted to their native ecological niche, and hybrids having higher fitness than parents within the transitional climate (bounded hybrid superiority model). Indeed, this model explained the maintenance of the hybrid zone and species integrity between hybridizing spruce species pairs from western North America (i.e., *Picea sitchensis *× *Picea glauca*, Hamilton et al., 2013a; Hamilton, Lexer, & Aitken, 2013b; Hamilton & Aitken, 2013; and *Picea engelmannii × P. glauca*, De La Torre, Wang, Jacquish, & Aitken, 2014; De La Torre et al., 2015). However, a closer look at the sympatric zone of *P. mariana* and *P. rubens*, where contemporary interspecific gene flow actually occurs, tells a different story. While an association between genetic ancestry and environmental variables should be expected if exogenous selection is a key process, temperature did not vary significantly among the three genotypic classes (*P. mariana* purebreds, hybrids, and *P. rubens* purebreds) within the sympatric zone. Hence, unlike the pattern found for hybridizing spruce species pairs of western North America (De La Torre et al., 2014, 2015; Hamilton & Aitken, 2013; Hamilton et al., 2013a,b), there was no significant relationship between hybrid index and temperature within the sympatric zone of black spruce and red spruce. By contrast, such a relationship was found between the hybrid index and precipitation, albeit with very low explanatory power. Precipitation was higher for *P. rubens* purebreds than for hybrids and *P. mariana* purebreds. Interestingly, this apparent association collapses when considered in light of the directional bias in interspecific gene flow. Indeed, within the sympatric zone, a difference in total annual amount of precipitation was only observed between red spruce and hybrids; yet, this cannot stand out as a strong selective filter because the gene flow was invariably high between these two genotypic classes. Furthermore, while an actual barrier to interspecific gene flow was strictly found between *P. mariana* and hybrids, the amount of precipitation did not differ between these.

While formally disentangling the role of environmental and genetic factors in the maintenance of the hybrid zone between black spruce and red spruce was beyond the scope of this study, uncovering directional biases in interspecific gene flow did provide some useful insights. Specifically, it allowed us to falsify an apparent association between hybrid index and precipitation we initially detected before considering asymmetrical patterns of introgression. Although we cannot rigorously discard the possibility of exogenous selection based solely on our exploratory analysis, we found no evidence that two of the most important climatic factors, namely temperature and precipitation, played a key role in the maintenance of species boundary between black spruce and red spruce. This is in sharp contrast with other hybridizing spruce species pairs from western North America for which temperature and precipitation determined the distribution and structure of hybrid zones and where climatic differences were greater than in our study (De La Torre et al., 2014, 2015; Hamilton & Aitken, 2013; Hamilton et al., 2013a,b). However, it must be acknowledged that there could be other exogenous selective pressures that were not assessed in the present study, such that a different ecological factor is more important than precipitation or temperature for preventing black spruce backcrosses. Hypothetically, assuming that (1) there is no reproductive phenological barrier, (2) selection (rather than purely neutral processes) is the primary mechanism implicated in determining contemporary patterns of introgression, and (3) exogenous selection is not a key determinant for maintaining the spruce hybrid zone, endogenous selective pressures (such as Bateson–Dobzhansky–Muller incompatibilities) could perhaps represent another asymmetrical mechanism preventing ongoing backcrossing toward black spruce at impermeable genomic regions. These genomic incompatibilities would be asymmetrical as backcrossing toward red spruce remains virtually unimpeded. It is well established that such asymmetrical incompatibilities constitute a possible outcome of the Bateson–Dobzhansky–Muller models (e.g., when unidirectionally inherited markers are involved in the incompatibility) and represent Darwin's corollary to Haldane's rule (Turelli & Moyle, 2007; Welch, 2004; Wu & Beckenbach, 1983).

In a previous study, we uncovered the semipermeable nature of the barriers to gene flow between black spruce and red spruce by identifying loci representing impermeable, neutral, and highly permeable genomic regions (de Lafontaine et al., 2015). With the present study, we moved forward our understanding about species delimitation of these progenitor‐derivative taxa by showing that the barriers to gene flow are asymmetrical. First, we suggested that the higher rate of gene flow from black spruce to red spruce than the opposite likely reflects the known long‐term demographic history of the two spruce taxa. Second, while backcrossing toward red spruce was invariably high across the genome, the actual species boundary is between hybrids and black spruce, where gene flow is impeded at those genomic regions impermeable to introgression. Hence, in this progenitor‐derivative species pair, the barrier to interspecific gene flow is asymmetrical and prevents introgression of the derivative species (red spruce) back into the genomic background of the progenitor species (black spruce). Uncovering directional biases in interspecific gene flow provided some insights about the relative importance of the selective pressures maintaining the hybrid zone and the integrity of closely related species. Specifically, considering asymmetrical patterns of introgression allowed us to falsify an apparent association between hybrid index and precipitation. Although our study did not completely rule out the role of exogenous selection in maintaining the species boundary between red spruce and black spruce, it does however advise caution when inferring exogenous selection pressure from a given ecological variable based on environment–genetic associations alone.

## Conflict of Interest

None declared.

## References

[ece32682-bib-0001] Abbott, R. J. , Albach, D. , Ansell, S. , Arntzen, J. W. , Baird, S. J. E. , Bierne, N. , … Zinner, D . (2013). Hybridization and speciation. Journal of Evolutionary Biology, 26, 229–246. 23323997 10.1111/j.1420-9101.2012.02599.x

[ece32682-bib-0002] Abdo, Z. , Crandall, K. A. , & Joyce, P. (2004). Evaluating the performance of likelihood methods for detecting population structure and migration. Molecular Ecology, 13, 837–851. 15012759 10.1111/j.1365-294x.2004.02132.x

[ece32682-bib-0003] Anderson, E. (1949). Introgressive hybridization. New York, NY: Wiley.

[ece32682-bib-0004] Andrew, R. L. , Ostevik, K. L. , Ebert, D. P. , & Rieseberg, L. H. (2012). Adaptation with gene flow across the landscape in a dune sunflower. Molecular Ecology, 21, 2078–2091. 22429200 10.1111/j.1365-294X.2012.05454.x

[ece32682-bib-0005] Arnold, M. L. (1997). Natural hybridization and evolution. Oxford, UK: Oxford University Press.

[ece32682-bib-0006] Arnold, M. L. , Tang, S. , Knapp, S. J. , & Martin, N. H. (2010). Asymmetric introgressive hybridization among Louisiana Iris species. Genes, 1, 9–22. 24710008 10.3390/genes1010009 PMC3960859

[ece32682-bib-0007] Baack, E. , Melo, M. C. , Rieseberg, L. H. , & Ortiz‐Barrientos, D. (2015). The origins of reproductive isolation in plants. New Phytologist, 207, 968–984. 25944305 10.1111/nph.13424

[ece32682-bib-0008] Bacilieri, R. , Ducousso, A. , Petit, R. J. , & Kremer, A. (1996). Mating system and asymmetric hybridization in a mixed stand of European oaks. Evolution, 50, 900–908. 28568948 10.1111/j.1558-5646.1996.tb03898.x

[ece32682-bib-0009] Barton, N. H. , & Hewitt, G. M. (1985). Analysis of hybrid zones. Annual Review of Ecology and Systematics, 16, 113–148.

[ece32682-bib-0010] Beerli, P. (2009). How to use MIGRATE or why are Markov chain Monte Carlo programs difficult to use? In Bertorelle G., Bruford M. W., Hauffe H. C., Rizzoli A., & Vernesi C. (Eds.), Population genetics for animal conservation (pp. 42–79). Cambridge, UK: Cambridge University Press.

[ece32682-bib-0011] Beerli, P. , & Felsenstein, J. (1999). Maximum‐likelihood estimation of migration rates and effective population numbers in two populations using a coalescent approach. Genetics, 152, 763–773. 10353916 10.1093/genetics/152.2.763 PMC1460627

[ece32682-bib-0012] Beerli, P. , & Felsenstein, J. (2001). Maximum likelihood estimation of a migration matrix and effective population sizes in n subpopulations using a coalescent approach. Proceedings of the National Academy of Sciences USA, 98, 4563–4568. 10.1073/pnas.081068098 PMC31874 11287657

[ece32682-bib-0013] Beerli, P. , & Palczewski, M. (2010). Unified framework to evaluate panmixia and migration direction among multiple sampling locations. Genetics, 185, 313–326. 20176979 10.1534/genetics.109.112532 PMC2870966

[ece32682-bib-0014] Benjamini, Y. , & Yekutieli, D. (2001). The control of the false discovery rate in multiple testing under dependency. Annals of Statistics, 29, 1165–1188.

[ece32682-bib-0015] Bobola, M. S. , Eckert, R. T. , Klein, A. S. , Stapelfeldt, K. , Hillenberg, K. A. , & Gendreau, S. B. (1996). Hybridization between *Picea rubens* and *Picea mariana*: Differences observed between montane and coastal island populations. Canadian Journal of Forest Research, 26, 444–452.

[ece32682-bib-0016] Burgess, K. S. , Morgan, M. , Deverno, L. , & Husband, B. C. (2005). Asymmetrical introgression between two *Morus* species (*M. alba*,* M. rubra*) that differ in abundance. Molecular Ecology, 14, 3471–3483. 16156816 10.1111/j.1365-294X.2005.02670.x

[ece32682-bib-0017] Carney, S. E. , Cruzan, M. B. , & Arnold, M. L. (1994). Reproductive interactions between hybridizing irises: Analyses of pollen‐tube growth and fertilization success. American Journal of Botany, 81, 1169–1175.

[ece32682-bib-0018] Currat, M. , Ruedi, M. , Petit, R. J. , & Excoffier, L. (2008). The hidden side of invasions: Massive introgression by local genes. Evolution, 62, 1908–1920. 18452573 10.1111/j.1558-5646.2008.00413.x

[ece32682-bib-0101] de Lafontaine, G. , Prunier, J. , Gérardi, S. , & Bousquet, J. (2015). Tracking the progression of speciation: Variable patterns of introgression across the genome provide insights on the species delimitation between progenitor‐derivative spruces (*Picea mariana × P. rubens*). Molecular Ecology, 24, 5229–5247. 26346701 10.1111/mec.13377

[ece32682-bib-0019] De La Torre, A. , Ingvarsson, P. K. , & Aitken, S. N. (2015). Genetic architecture and genomic patterns of gene flow between hybridizing species of *Picea* . Heredity, 115, 153–164. 25806545 10.1038/hdy.2015.19 PMC4815442

[ece32682-bib-0020] De La Torre, A. , Wang, T. , Jacquish, B. , & Aitken, S. N. (2014). Adaptation and exogenous selection in a *Picea glauca* ×* Picea engelmannii* hybrid zone: Implications for forest management under climate change. New Phytologist, 201, 687–699. 24200028 10.1111/nph.12540 PMC4285121

[ece32682-bib-0021] Field, D. L. , Ayre, D. J. , Whelan, R. J. , & Young, A. G. (2011). Patterns of hybridization and asymmetrical gene flow in hybrid zones of the rare *Eucalyptus aggregata* and common *E. rubida* . Heredity, 106, 841–853. 21063438 10.1038/hdy.2010.127 PMC3186239

[ece32682-bib-0022] Fitzpatrick, B. M. , Johnson, J. R. , Kump, D. K. , Smith, J. J. , Voss, S. R. , & Shaffer, H. B. (2010). Rapid spread of invasive genes into a threatened native species. Proceedings of the National Academy of Sciences USA, 107, 3606–3610. 10.1073/pnas.0911802107 PMC2840512 20133596

[ece32682-bib-0023] Gordon, A. G. (1976). The taxonomy and genetics of *Picea rubens* and its relationship to *Picea mariana* . Canadian Journal of Botany, 54, 781–813.

[ece32682-bib-0024] Guichoux, E. , Garnier‐Géré, P. , Lagache, L. , Lang, T. , Boury, C. , & Petit, R. J. (2013). Outlier loci highlight the direction of introgression in oaks. Molecular Ecology, 22, 450–462. 23190431 10.1111/mec.12125

[ece32682-bib-0025] Hamilton, J. A. , & Aitken, S. N. (2013). Genetic and morphological structure of a spruce hybrid (*Picea sitchensis* × *P. glauca*) zone along a climatic gradient. American Journal of Botany, 100, 1651–1662. 23935108 10.3732/ajb.1200654

[ece32682-bib-0026] Hamilton, J. A. , Lexer, C. , & Aitken, S. N. (2013a). Differential introgression reveals candidate genes for selection across a spruce (*Picea sitchensis* × *P. glauca*) hybrid zone. New Phytologist, 197, 927–938. 23228022 10.1111/nph.12055

[ece32682-bib-0027] Hamilton, J. A. , Lexer, C. , & Aitken, S. N. (2013b). Genomic and phenotypic architecture of a spruce hybrid zone (*Picea sitchensis* × *P. glauca*). Molecular Ecology, 22, 827–841. 22967172 10.1111/mec.12007

[ece32682-bib-0028] Harrison, R. G. , & Larson, E. L. (2014). Hybridization, introgression, and the nature of species boundaries. Journal of Heredity, 105, 795–809. 25149255 10.1093/jhered/esu033

[ece32682-bib-0029] Jaramillo‐Correa, J. P. , & Bousquet, J. (2003). New evidence from mitochondrial DNA of a progenitor‐derivative species relationship between black spruce and red spruce (Pinaceae). American Journal of Botany, 90, 1801–1806. 21653356 10.3732/ajb.90.12.1801

[ece32682-bib-0030] Kane, N. C. , King, M. G. , Barker, M. S. , Raduski, A. , Karrenberg, S. , Yatabe, Y. , … Rieseberg, L. H. (2009). Comparative genomic and population genetic analyses indicate highly porous genomes and high levels of gene flow between divergent *Helianthus* species. Evolution, 63, 2061–2075. 19473382 10.1111/j.1558-5646.2009.00703.x PMC2731706

[ece32682-bib-0031] Keim, P. , Paige, K. N. , Whitham, T. G. , & Lark, K. G. (1989). Genetic analysis of an interspecific hybrid swarm of *Populus*: Occurrence of unidirectional introgression. Genetics, 123, 557–565. 2574697 10.1093/genetics/123.3.557 PMC1203828

[ece32682-bib-0032] Key, K. H. L. (1968). The concept of stasipatric speciation. Systematic Biology, 17, 14–22.

[ece32682-bib-0034] Lepais, O. , Petit, R. J. , Guichoux, E. , Lavabre, J. E. , Alberto, F. , Kremer, A. , & Gerber, S. (2009). Species relative abundance and direction of introgression in oaks. Molecular Ecology, 18, 2228–2242. 19302359 10.1111/j.1365-294X.2009.04137.x

[ece32682-bib-0035] Lexer, C. , Fay, M. F. , Joseph, J. A. , Nica, M.‐S. , & Heinze, B. (2005). Barrier to gene flow between two ecologically divergent *Populus* species, *P. alba* (white poplar) and *P. tremula* (European aspen): The role of ecology and life history in gene introgression. Molecular Ecology, 14, 1045–1057. 15773935 10.1111/j.1365-294X.2005.02469.x

[ece32682-bib-0036] Lexer, C. , Joseph, J. A. , van Loo, M. , Barbará, T. , Fay, M. F. , & Buerkle, C. A. (2010). Genomic admixture analysis in European *Populus* spp. reveals unexpected patterns of reproductive isolation and mating. Genetics, 186, 699–712. 20679517 10.1534/genetics.110.118828 PMC2954470

[ece32682-bib-0037] Lexer, C. , & Widmer, A. (2008). The genic view of plant speciation: Recent progress and emerging questions. Philosophical Transactions of the Royal Society B, 363, 3023–3036. 10.1098/rstb.2008.0078 PMC2607315 18579476

[ece32682-bib-0038] Lindbladh, M. , Jacobson, G. L. Jr , & Schauffler, M. (2003). The postglacial history of three *Picea* species in New England, USA. Quaternary Research, 59, 61–69.

[ece32682-bib-0039] Lowry, D. B. , Modliszewski, J. L. , Wright, K. M. , Wu, C. A. , & Willis, J. H. (2008). The strength and genetic basis of reproductive isolating barriers in flowering plants. Philosophical Transactions of the Royal Society B, 363, 3009–3021. 10.1098/rstb.2008.0064 PMC2607309 18579478

[ece32682-bib-0040] Luttikhuizen, P. C. , Drent, J. , Peijnenburg, K. T. C. A. , van der Veer, H. W. , & Johannesson, K. (2012). Genetic architecture in a marine hybrid zone: Comparing outlier detection and genomic clines analysis in the bivalve *Macoma balthica* . Molecular Ecology, 21, 3048–3061. 22554266 10.1111/j.1365-294X.2012.05586.x

[ece32682-bib-0041] Major, J. E. , Barsi, D. C. , Mosseler, A. , Campbell, M. , & Rajora, O. P. (2003). Light‐energy processing and freezing‐tolerance traits in red spruce and black spruce: Species and seed‐source variation. Tree Physiology, 23, 685–694. 12777241 10.1093/treephys/23.10.685

[ece32682-bib-0042] Major, J. E. , Mosseler, A. , Barsi, D. C. , Campbell, M. , & Rajora, O. P. (2003a). Morphometric, allometric, and developmentally adaptive traits in red spruce and black spruce. I. Species and seed‐source variation. Canadian Journal of Forest Research, 33, 885–896.

[ece32682-bib-0043] Major, J. E. , Mosseler, A. , Barsi, D. C. , Campbell, M. , & Rajora, O. P. (2003b). Morphometric, allometric, and developmentally adaptive traits in red spruce and black spruce. II. Seedling and mature tree assessment of controlled intra‐ and inter‐specific hybrids. Canadian Journal of Forest Research, 33, 897–909.

[ece32682-bib-0044] Major, J. E. , Mosseler, A. , Johnsen, K. H. , Rajora, O. P. , Barsi, D. C. , Kim, K.‐H. , … Campbell, M. (2005). Reproductive barriers and hybridity in two spruces, *Picea rubens* and *Picea mariana*, sympatric in eastern North America. Canadian Journal of Botany, 83, 163–175.

[ece32682-bib-0045] Manley, S. A. M. (1972). The occurrence of hybrid swarms of red and black spruces in central New Brunswick. Canadian Journal of Forest Research, 2, 381–391.

[ece32682-bib-0046] Manley, S. A. M. , & Ledig, F. T. (1979). Photosynthesis in black and red spruce and their hybrid derivatives: Ecological isolation and hybrid adaptive inferiority. Canadian Journal of Botany, 57, 305–314.

[ece32682-bib-0047] Martin, N. H. , Bouck, A. C. , & Arnold, M. L. (2006). Detecting adaptive trait introgression between *Iris fulva* and *I. brevicaulis* in highly selective field conditions. Genetics, 172, 2481–2489. 16415358 10.1534/genetics.105.053538 PMC1456367

[ece32682-bib-0048] Martinsen, G. D. , Whitham, T. G. , Turek, R. J. , & Keim, P. (2001). Hybrid populations selectively filter gene introgression between species. Evolution, 55, 1325–1335. 11525457 10.1111/j.0014-3820.2001.tb00655.x

[ece32682-bib-0049] Minder, A. M. , & Widmer, A. (2008). A population genomic analysis of species boundaries: Neutral processes, adaptive divergence and introgression between two hybridizing plant species. Molecular Ecology, 17, 1552–1563. 18321255 10.1111/j.1365-294X.2008.03709.x

[ece32682-bib-0050] Moore, W. S. (1977). An evaluation of narrow hybrid zones in vertebrates. Quarterly Review of Biology, 52, 263–277.

[ece32682-bib-0051] Morgenstern, E. K. , & Farrar, J. L. (1964). Natural hybridization in red spruce and black spruce. Technical Report No. 4, University of Toronto, Toronto, Canada.

[ece32682-bib-0052] Nielsen, E. E. , Bach, L. A. , & Kotlick, P. (2006). HYBRIDLAB (version 1.0): A program for generating simulated hybrids from population samples. Molecular Ecology Notes, 6, 971–973.

[ece32682-bib-0053] Nolte, A. W. , Gompert, Z. , & Buerkle, C. A. (2009). Variable patterns of introgression in two sculpin hybrid zones suggest that genomic isolation differs among populations. Molecular Ecology, 18, 2615–2627. 19457191 10.1111/j.1365-294X.2009.04208.x

[ece32682-bib-0054] Nosil, P. (2012). Ecological speciation. Oxford, UK: Oxford University Press.

[ece32682-bib-0055] Ortego, J. , Gugger, P. F. , & Sork, V. L. (2016). Impacts of human‐induced environmental disturbances on hybridization between two ecologically differentiated Californian oak species. New Phytologist, doi: 10.1111/nph.14182. 10.1111/nph.14182 27621132

[ece32682-bib-0056] Perron, M. , & Bousquet, J. (1997). Natural hybridization between black spruce and red spruce. Molecular Ecology, 6, 725–734.

[ece32682-bib-0057] Perron, M. , Perry, D. J. , Andalo, C. , & Bousquet, J. (2000). Evidence from sequence‐tagged‐site markers of a recent progenitor‐derivative species pair in conifers. Proceedings of the National Academy of Sciences USA, 97, 11331–11336. 10.1073/pnas.200417097 PMC17200 11016967

[ece32682-bib-0058] Petit, R. J. , Bodénès, C. , Ducousso, A. , Roussel, G. , & Kremer, A. (2003). Hybridization as a mechanism of invasion in oaks. New Phytologist, 161, 151–164.

[ece32682-bib-0059] Pritchard, J. K. , Stephens, M. , & Donnelly, P. (2000). Inference of population structure using multilocus genotype data. Genetics, 155, 945–959. 10835412 10.1093/genetics/155.2.945 PMC1461096

[ece32682-bib-0060] Prunier, J. , Gérardi, S. , Laroche, J. , Beaulieu, J. , & Bousquet, J. (2012). Parallel and lineage‐specific molecular adaptation to climate in boreal black spruce. Molecular Ecology, 21, 4270–4286. 22805595 10.1111/j.1365-294X.2012.05691.x

[ece32682-bib-0061] Régnière, J. (1996). Generalized approach to landscape‐wide seasonal forecasting with temperature‐driven simulation models. Environmental Entomology, 25, 869–881.

[ece32682-bib-0062] Rhymer, J. M. , & Simberloff, D. (1996). Extinction by hybridization and introgression. Annual Review of Ecology and Systematics, 27, 83–109.

[ece32682-bib-0063] Rieseberg, L. H. , & Blackman, B. K. (2010). Speciation genes in plants. Annals of Botany, 106, 439–455. 20576737 10.1093/aob/mcq126 PMC2924826

[ece32682-bib-0064] Rieseberg, L. H. , Whitton, J. , & Gardner, K. (1999). Hybrid zones and the genetic architecture of a barrier to gene flow between two sunflower species. Genetics, 152, 713–727. 10353912 10.1093/genetics/152.2.713 PMC1460641

[ece32682-bib-0065] Scascitelli, M. , Whitney, K. D. , Randell, R. A. , King, M. G. , Buerkle, C. A. , & Rieseberg, L. H. (2010). Genome scan of hybridizing sunflowers from Texas (*Helianthus annuus* and *H. debilis*) reveals asymmetric patterns of introgression and small islands of genomic differentiation. Molecular Ecology, 19, 521–541. 20355258 10.1111/j.1365-294x.2009.04504.x

[ece32682-bib-0066] Scotti‐Saintagne, C. , Mariette, S. , Porth, I. , Goicoechea, P. G. , Barreneche, T. , Bodénès, C. , … Kremer, A. (2004). Genome scanning for interspecific differentiation between two closely related oak species [*Quercus robur* L. and *Q. petraea* (Matt.) Liebl.]. Genetics, 168, 1615–1626. 15579711 10.1534/genetics.104.026849 PMC1448783

[ece32682-bib-0067] Starr, T. N. , Gadek, K. E. , Yoder, J. B. , Flatz, R. , & Smith, C. I. (2013). Asymmetric hybridization and gene flow between Joshua trees (Agavaceae: *Yucca*) reflect differences in pollinator host specificity. Molecular Ecology, 22, 437–449. 23190404 10.1111/mec.12124

[ece32682-bib-0068] Strasburg, J. L. , Sherman, N. A. , Wright, K. M. , Moyle, L. C. , Willis, J. H. , & Rieseberg, L. H. (2012). What can patterns of differentiation across plant genomes tell us about adaptation and speciation? Philosophical Transactions of the Royal Society B, 367, 364–373. 10.1098/rstb.2011.0199 PMC3233712 22201166

[ece32682-bib-0069] Sweigart, A. L. , & Willis, J. H. (2003). Patterns of nucleotide diversity in two species of *Mimulus* are affected by mating system and asymmetric introgression. Evolution, 57, 2490–2506. 14686526 10.1111/j.0014-3820.2003.tb01494.x

[ece32682-bib-0070] Tiffin, P. , Olson, M. S. , & Moyle, L. C. (2001). Asymmetrical crossing barriers in angiosperms. Proceedings of the Royal Society of London Series B, 268, 861–867. 11345333 10.1098/rspb.2000.1578 PMC1088681

[ece32682-bib-0071] Turelli, M. , & Moyle, L. C. (2007). Asymmetric postmating isolation: Darwin's corollary to Haldane's rule. Genetics, 176, 1059–1088. 17435235 10.1534/genetics.106.065979 PMC1894575

[ece32682-bib-0072] Vähä, J.‐P. , & Primmer, C. R. (2006). Efficiency of model‐based Bayesian methods for detecting hybrid individuals under different hybridization scenarios and with a different numbers of loci. Molecular Ecology, 15, 63–72. 16367830 10.1111/j.1365-294X.2005.02773.x

[ece32682-bib-0073] Welch, J. J. (2004). Accumulating Dobzhansky‐Muller incompatibilities: Reconciling theory and data. Evolution, 58, 1145–1156. 15266965 10.1111/j.0014-3820.2004.tb01695.x

[ece32682-bib-0074] Wu, C.‐I. (2001). The genic view of the process of speciation. Journal of Evolutionary Biology, 14, 851–865.

[ece32682-bib-0075] Wu, C.‐I. , & Beckenbach, A. T. (1983). Evidence for extensive genetic differentiation between the sex‐ratio and the standard arrangement of *Drosophila pseudoobscura* and *D. persimilis* and identification of hybrid sterility factors. Genetics, 105, 71–96. 17246158 10.1093/genetics/105.1.71 PMC1202152

