# Supplementary material for: Asymmetry matters: A genomic assessment of directional biases in gene flow between hybridizing spruces
Source: Ecol Evol. 2017 Apr 22;7(11):3883–93. doi: 10.1002/ece3.2682 (PMC5468134; doi:10.1002/ece3.2682)
Supplement: Supplementary file 1 [file ECE3-7-3883-s001.docx]

**SUPPORTING INFORMATION**

**Asymmetry matters: a genomic assessment of directional biases in gene flow between hybridizing spruces**

Guillaume de Lafontaine and Jean Bousquet

**Table S1. Geographic location, sample size (*N*), and local climatic variables for 33 allopatric and sympatric populations *Picea mariana* and *Picea rubens*.**

| Geographic zone | Location | Province or State | *N* | Latitude  (°N) | Longitude  (°W) | Elevation  (m a.s.l.) | Mean annual temperature  (ºC) | Total annual precipitation  (mm) |
| --- | --- | --- | --- | --- | --- | --- | --- | --- |
| Black spruce allopatric | Long Point Road | QC | 10 | 54.0 | 79.1 | 2 | -2.6 | 711.8 |
|  | Waskaganish | QC | 12 | 51.5 | 78.8 | 11 | -0.6 | 800.0 |
|  | Manicouagan | QC | 7 | 50.4 | 68.5 | 420 | -0.3 | 1001.1 |
|  | Parc Mistassini | QC | 10 | 50.3 | 73.4 | 433 | -0.7 | 956.5 |
|  | Chibougamau | QC | 13 | 49.2 | 73.6 | 380 | 0.0 | 962.8 |
|  | Condé | QC | 14 | 49.1 | 72.7 | 295 | 0.6 | 935.2 |
|  | Ligneris | QC | 13 | 48.9 | 78.5 | 316 | 0.5 | 898.8 |
|  | Parc de la Vérendrye | QC | 5 | 47.1 | 74.6 | 371 | 2.5 | 1016.3 |
|  | St-Michel-des-Saints | QC | 8 | 46.8 | 74.4 | 386 | 2.7 | 951.7 |
| Sympatric | Tourville | QC | 12 | 47.1 | 70.0 | 274 | 3.2 | 1110.8 |
|  | Laurier Station | QC | 9 | 46.3 | 71.4 | 110 | 4.3 | 1150.6 |
|  | Villeroy | QC | 11 | 46.2 | 71.5 | 117 | 4.2 | 1127.1 |
|  | Black Lake | QC | 12 | 45.6 | 71.2 | 350 | 4.2 | 1276.4 |
|  | St-Gabriel-de-Valcartier | QC | 12 | 46.5 | 71.3 | 202 | 3.5 | 1336.8 |
|  | St-Raymond | QC | 13 | 46.5 | 71.5 | 145 | 3.8 | 1317.2 |
|  | St-Louis de Blandford | QC | 14 | 46.1 | 72.0 | 130 | 4.9 | 1104.9 |
|  | St-Méthode Frontenac | QC | 12 | 46.0 | 71.0 | 360 | 4.0 | 1149.0 |
|  | La Patrie | QC | 12 | 45.2 | 71.1 | 471 | 4.0 | 1276.3 |
|  | West Ditton | QC | 14 | 45.2 | 71.2 | 456 | 4.0 | 1283.8 |
|  | Lac Trois-Saumons | QC | 13 | 47.1 | 70.1 | 450 | 2.3 | 1187.8 |
|  | Ste-Anne-de-Beaupré | QC | 11 | 47.0 | 70.6 | 110 | 4.3 | 1212.5 |
|  | Duschenay | QC | 6 | 46.9 | 71.7 | 192 | 3.4 | 1345.4 |
|  | Lauzon | QC | 14 | 46.4 | 71.1 | 140 | 4.4 | 1191.1 |
|  | Fredericton | NB | 13 | 46.0 | 66.4 | 112 | 4.9 | 1201.8 |
|  | Estrie | QC | 16 | 45.2 | 71.1 | 472 | 4.0 | 1281.6 |
|  | St-Charles de Mandeville | QC | 1 | 46.3 | 75.2 | 155 | 4.5 | 1028.2 |
| Red spruce allopatric | Upper Jay | NY | 12 | 44.4 | 73.7 | 222 | 6.9 | 994.8 |
|  | October MSF | MA | 24 | 42.4 | 73.2 | 454 | 6.9 | 1232.8 |
|  | Bear Meadows | PA | 4 | 40.7 | 77.7 | 500 | 9.3 | 1059.8 |
|  | Glade Run | WV | 17 | 38.6 | 79.8 | 944 | 8.0 | 1246.6 |
|  | Indian Cap | TN | 11 | 35.6 | 83.5 | 1254 | 9.4 | 1544.5 |
|  | NewFound Gap | NC | 15 | 35.5 | 83.4 | 1200 | 9.8 | 1571.3 |
|  | Blue Ridge | NC | 15 | 35.7 | 82.3 | 1450 | 9.4 | 1420.2 |

Abbreviations: QC, Québec; NB, New Brunswick; NY, New York; MA, Massachusetts; PA, Pennsylvania; WV, West Virginia; TN, Tennessee; NC, North Carolina.

| Impermeable markers (*N* = 23) | | | | | | | | | | |
| --- | --- | --- | --- | --- | --- | --- | --- | --- | --- | --- |
| Parameter | Run1 | Run2 | Run3 | Run4 | Run5 | Run6 | Run7 | Run8 | Run9 | Run10 |
| Θ*_P.mariana_* | 0.37 (0.36-0.39) | 0.41 (0.38-0.43) | 0.29 (0.28-0.30) | 0.31 (0.30-0.33) | 0.26 (0.25-0.28) | 0.26 (0.25-0.27) | 0.28 (0.27-0.30) | 0.40 (0.38-0.42) | 0.41 (0.39-0.43) | 0.26 (0.25-0.28) |
| Θ_Hybrids_ | 0.13 (0.12-0.13) | 0.07 (0.07-0.07) | 0.12 (0.12-0.13) | 0.14 (0.13-0.15) | 0.12 (0.11-0.12) | 0.11 (0.10-0.11) | 0.12 (0.11-0.13) | 0.14 (0.13-0.15) | 0.21 (0.20-0.22) | 0.12 (0.12-0.13) |
| Θ*_P.rubens_* | 0.01 (0.01-0.02) | 0.01 (0.01-0.01) | 0.02 (0.02-0.02) | 0.01 (0.01-0.02) | 0.01 (0.01-0.01) | 0.01 (0.01-0.01) | 0.02 (0.01-0.02) | 0.01 (0.01-0.01) | 0.01 (0.01-0.01) | 0.01 (0.01-0.01) |
| *M*_Hybrids→_*_P.mariana_* | 0.76 (0.64-0.90) | 0.92 (0.76-1.09) | 1.42 (1.22-1.65) | 0.79 (0.60-1.01) | 0.61 (0.46-0.79) | 0.30 (0.22-0.39) | 1.02 (0.81-1.26) | 1.08 (0.86-1.32) | 0.47 (0.39-0.57) | 0.77 (0.59-1.00) |
| *M_P.rubens_*_→_*_P.mariana_* | 0.44 (0.35-0.54) | 0.52 (0.41-0.65) | 0.71 (0.57-0.88) | 0.74 (0.56-0.96) | 1.05 (0.84-1.29) | 0.37 (0.28-0.47) | 0.91 (0.72-1.14) | 0.35 (0.24-0.49) | 0.21 (0.15-0.27) | 0.42 (0.29-0.59) |
| *M_P.mariana_*_→Hybrids_ | 1.27 (1.07-1.49) | 1.81 (1.50-2.15) | 10.21 (9.02-11.49) | 6.56 (5.45-7.82) | 1.98 (1.57-2.45) | 1.53 (1.25-1.85) | 2.86 (2.36-3.44) | 2.85 (2.25-3.54) | 0.40 (0.28-0.51) | 2.46 (1.92-3.10) |
| *M_P.rubens_*_→Hybrids_ | 9.82 (9.25-10.40) | 7.88 (7.22-8.58) | 43.43 (40.93-46.02) | 42.29 (39.27-45.45) | 27.75 (26.12-29.45) | 18.40 (17.38-19.46) | 12.33 (11.28-13.44) | 28.87 (26.90-30.94) | 8,57 (8.12-9.03) | 32.95 (30.80-35.21) |
| *M_P.mariana_*_→_*_P.rubens_* | 1.64 (1.33-1.99) | 2.36 (1.90-2.90) | 0.87 (0.67-1.10) | 2.48 (1.98-3.06) | 1.88 (1.43-2.41) | 0.60 (0.46-0.78) | 3.15 (2.45-3.98) | 0.98 (0.69-1.34) | 1.34 (1.04-1.71) | 1.16 (0.83-1.57) |
| *M*_Hybrids→_*_P.rubens_* | 17.36 (16.32-18.46) | 11.54 (10.46-12.72) | 13.40 (12.57-14.27) | 17.55 (16.14-19.04) | 29.00 (27.06-31.04) | 11.48 (10.80-12.19) | 21.19 (19.25-23.25) | 19.20 (17.77-20.70) | 26.37 (24.90-27.89) | 22.05 (20.47-23.71) |
| Neutral markers (*N* = 238) | | | | | | | | | | |
| Parameter | Run1 | Run2 | Run3 | Run4 | Run5 | Run6 | Run7 | Run8 | Run9 | Run10 |
| Θ*_P.mariana_* | 0.43 (0.42-0.43) | 0.27 (0.27-0.28) | 0.28 (0.28-0.29) | 0.31 (0.30-0.31) | 0.38 (0.37-0.38) | 0.29 (0.28-0.29) | 0.34 (0.33-0.34) | 0.57 (0.56-0.58) | 0.35 (0.35-0.36) | 0.36 (0.36-0.37) |
| Θ_Hybrids_ | 0.05 (0.05-0.05) | 0.05 (0.05-0.05) | 0.05 (0.05-0.05) | 0.05 (0.05-0.05) | 0.05 (0.05-0.05) | 0.05 (0.05-0.05) | 0.05 (0.05-0.05) | 0.05 (0.05-0.05) | 0.05 (0.05-0.05) | 0.04 (0.04-0.04) |
| Θ*_P.rubens_* | 0.01 (0.01-0.01) | 0.01 (0.01-0.01) | 0.01 (0.01-0.01) | 0.02 (0.02-0.02) | 0.01 (0.01-0.01) | 0.01 (0.01-0.01) | 0.01 (0.01-0.01) | 0.01 (0.01-0.01) | 0.01 (0.01-0.01) | 0.01 (0.01-0.01) |
| *M*_Hybrids→_*_P.mariana_* | 5.31 (5.13-5.50) | 6.13 (5.86-6.41) | 4.97 (4.74-5.20) | 5.65 (5.44-5.87) | 5.00 (4.80-5.21) | 6.12 (5.84-6.41) | 5.39 (5.17-5.61) | 3.65 (3.50-3.79) | 4.64 (4.43-4.86) | 4.35 (4.14-4.56) |
| *M_P.rubens_*_→_*_P.mariana_* | 3.03 (2.90-3.18) | 5.30 (5.05-5.56) | 4.67 (4.45-4.90) | 3.51 (3.35-3.68) | 3.33 (3.17-3.50) | 5.64 (5.38-5.92) | 3.66 (3.47-3.84) | 1.95 (1.84-2.05) | 2.53 (2.38-2.69) | 3.16 (2.99-3.34) |
| *M_P.mariana_*_→Hybrids_ | 8.80 (8.43-9.17) | 11.89 (11.27-12.54) | 10.44 (9.90-10.99) | 10.81 (10.35-11.29) | 17.79 (17.00-18.59) | 13.89 (13.15-14.65) | 17.39 (16.63-18.18) | 6.04 (5.71-6.39) | 12.42 (11.76-13.10) | 17.12 (16.23-18.05) |
| *M_P.rubens_*_→Hybrids_ | 29.28 (28.60-29.96) | 29.45 (28.46-30.46) | 28.99 (28.09-29.90) | 23.44 (22.75-24.13) | 27.43 (26.45-28.44) | 33.77 (32.60-34.97) | 29.18 (28.17-30.22) | 24.54 (23.86-25.24) | 28.73 (27.69-29.79) | 25.98 (24.87-27.13) |
| *M_P.mariana_*_→_*_P.rubens_* | 5.23 (4.96-5.52) | 10.82 (10.18-11.48) | 8.45 (7.92-9.00) | 7.60 (7.17-8.04) | 9.03 (8.50-9.59) | 13.85 (13.00-14.73) | 7.17 (6.74-7.62) | 4.31 (4.00-4.63) | 9.67 (8.98-10.40) | 7.18 (6.64-7.75) |
| *M*_Hybrids→_*_P.rubens_* | 28.02 (27.38-28.68) | 26.96 (25.93-28.02) | 31.25 (30.21-32.32) | 25.21 (24.43-26.01) | 19.72 (18.92-20.55) | 31.93(30.64-33.27) | 17.38 (16.69-18.10) | 27.52 (26.73-28.32) | 26.46 (25.29-27.67) | 18.72 (17.83-19.64) |
| Highly permeable markers (*N* = 39) | | | | | | | | | | |
| Parameter | Run1 | Run2 | Run3 | Run4 | Run5 | Run6 | Run7 | Run8 | Run9 | Run10 |
| Θ*_P.mariana_* | 0.18 (0.17-0.18) | 0.16 (0.15-0.16) | 0.13 (0.12-0.13) | 0.12 (0.11-0.12) | 0.10 (0.10-0.11) | 0.12 (0.12-0.13) | 0.14 (0.13-0.15) | 0.15 (0.15-0.16) | 0.14 (0.14-0.15) | 0.13 (0.12-0.13) |
| Θ_Hybrids_ | 0.07 (0.07-0.08) | 0.07 (0.07-0.08) | 0.09 (0.09-0.09) | 0.08 (0.08-0.08) | 0.09 (0.08-0.09) | 0.09 (0.09-0.10) | 0.08 (0.07-0.08) | 0.08 (0.08-0.08) | 0.09 (0.09-0.10) | 0.10 (0.10-0.11) |
| Θ*_P.rubens_* | 0.01 (0.01-0.01) | 0.02 (0.02-0.02) | 0.01 (0.01-0.01) | 0.01 (0.01-0.01) | 0.01 (0.01-0.01) | 0.01 (0.01-0.01) | 0.01 (0.01-0.01) | 0.01 (0.01-0.01) | 0.01 (0.01-0.01) | 0.01 (0.01-0.01) |
| *M*_Hybrids→_*_P.mariana_* | 22.28 (20.88-23.74) | 24.45 (22.51-26.50) | 24.23 (22.27-26.30) | 22.55 (20.41-24.85) | 29.15 (26.45-32.04) | 20.69 (27.99-33.55) | 24.58 (22.48-26.81) | 19.63 (18.0.1-21.35) | 28.87 (26.53-31.36) | 17.73 (16.11-19.45) |
| *M_P.rubens_*_→_*_P.mariana_* | 6.06 (5.35-6.84) | 18.18 (16.52-19.96) | 9.74 (8.55-11.04) | 15.46 (13.72-17.33) | 33.33 (30.37-36.48) | 27.23 (24.65-30.03) | 20.69 (18.79-22.72) | 8.64 (7.58-9.81) | 10.38 (9.05-11.83) | 7.47 (6.43-8.61) |
| *M_P.mariana_*_→Hybrids_ | 58.69 (55.03-62.51) | 41.14 (37.69-44.78) | 40.88 (37.63-44.34) | 28.80 (25.95-31.85) | 22.98 (20.78-25.33) | 29.24 (26.46-32.21) | 11.48 (10.44-12.58) | 24.61 (22.15-27.23) | 27.94 (25.56-30.47) | 28.51 (25.94-31.24) |
| *M_P.rubens_*_→Hybrids_ | 35.06 (32.21-38.07) | 31.27 (28.28-34.46) | 28.84 (26.13-31.74) | 29.43 (26.51-32.56) | 22.92 (20.73-25.26) | 26.90 (24.29-26.69) | 8.51 (7.64-9.45) | 23.08 (20.82-25.50) | 11.86 (10.30-13.62) | 29.42 (26.76-32.25) |
| *M_P.mariana_*_→_*_P.rubens_* | 11.80 (10.46-13.26) | 50.62 (45.20-56.44) | 24.63 (21.56-27.97) | 42.05 (36.52-48.05) | 20.69 (17.93-23.72) | 45.45 (39.88-51.52) | 9.18 (8.06-10.38) | 11.04 (9.13-13.17) | 10.80 (8.86-13.07) | 39.33 (34.04-45.12) |
| *M*_Hybrids→_*_P.rubens_* | 19.03 (17.31-20.85) | 50.45 (45.11-56.20) | 34.11 (30.41-38.12) | 47.47 (41.44-54.03) | 24.76 (21.72-28.07) | 40.64 (35.21-46.59) | 7.33 (6.39-8.36) | 38.39 (34.77-42.26) | 16.28 (13.90-18.97) | 51.16 (45.06-57.75) |

**Table S2. Maximum-likelihood estimates and 95% confidence intervals (in parentheses) of the mutation-scaled effective population size (Θ) and the mutation-scaled migration rate (*M*) parameters for each MIGRATE-N run.**
